# Optimization of Plant Production by Seed Treatment in Two Wild Subspecies of *Narcissus pseudonarcissus* Rich in Alkaloids

**DOI:** 10.3390/molecules25194439

**Published:** 2020-09-27

**Authors:** Raquel Herranz, Miguel A. Copete, José M. Herranz, Elena Copete, Pablo Ferrandis

**Affiliations:** 1 ETSIAM, Department of Plant Production and Agricultural Technology, University of Castilla-La Mancha, University Campus s/n, 02071 Albacete, Spain; raquel.herranz@alu.uclm.es (R.H.); jose.herranz@uclm.es (J.M.H.); mariaelena.copete@uclm.es (E.C.); pablo.ferrandis@uclm.es (P.F.); 2 Botanical Institute, University of Castilla-La Mancha, Avenida de la Mancha s/n, 02006 Albacete, Spain

**Keywords:** alkaloid, Amaryllidaceae, deep simple epicotyl morphophysiological dormancy, dormancy breakage, embryo growth, germination, intermediate complex morphophysiological dormancy, *Narcissus pseudonarcissus* L.

## Abstract

The daffodil *Narcissus pseudonarcissus* L. contains alkaloids of pharmaceutical interest. Wild daffodil populations have diverse genetic backgrounds and various genetic traits of possible importance. Developing protocols for plant production from seeds may ensure the availability of a large reservoir of individuals as well as being important for species with bulbs that are difficult to acquire. The closely related *Narcissus pseudonarcissus* subsp. *munozii-garmendiae* and subsp. *nevadensis* were investigated in this study because the alkaloids isolated from both are of high pharmacological interest. At the dispersal time, the seeds of both were dormant with underdeveloped embryos, i.e., morphophysiological dormancy (MPD). Experiments were conducted outdoors and under controlled laboratory conditions. Embryo growth and the percentages of radicle and seedling emergence were calculated under different temperature–light stratifications. In *N. munozii-garmendiae*, embryo growth occurred during warm stratification (28/14 °C or 25/10 °C) and the radicle then emerged when the temperature decreased, but the shoot was dormant. In *N. nevadensis*, the seeds germinated when cold stratified (5 °C) and then incubated at cool temperatures. Thus, *N. munozii-garmendiae* and *N. nevadensis* exhibit different levels of MPD, i.e., deep simple epicotyl and intermediate complex, respectively. Plant production protocols from seeds were established for both taxa in this study.

## 1. Introduction

Many species of *Narcissus* (Amaryllidaceae) contain small chemical molecules with various medicinal properties. The pharmaceutical potential of Amaryllidaceae alkaloids including galanthamine, lycorine, haemanthamine, and pancratistatin, is well recognized, primarily as anticancer drugs [1]. Recently, 21 known Amaryllidaceae alkaloids with various structural types and one previously undescribed alkaloid named narcimatuline were isolated from the fresh bulbs of *Narcissus pseudonarcissus* L. and identified as potential drugs for use in the treatment of Alzheimer’s disease [2].

Many previous studies of alkaloids from the Amaryllidaceae were conducted with ornamental *Narcissus* cultivars e.g., [2,3,4] because of their large-scale cultivation for the ornamental plant industry. By contrast, the alkaloids present in wild populations of daffodils have been analyzed less frequently e.g., [5,6,7,8]. Wild plant resources have great potential for supplying raw materials to the pharmaceutical industry but wild subspecies are usually unsuitable for agricultural exploitation because of limitations in terms of their availability or a lack of previous analyses. The limitation due to low resource availability can be minimized by seed collection and sexual reproduction of daffodils, and although little is known about the seed physiology in *Narcissus* [9], several studies have been published on this subject in recent years [10,11,12,13,14].

In a recent review of *Narcissus* systematics, Aedo [15] recognized the existence of 33 taxa (subspecies level) in the Iberian Peninsula, where 15 are endemic, thereby demonstrating the importance of this geographic area for the evolution of the genus. In the present study, we focused on section *Pseudonarcissus*, which includes nine taxa with seeds that contain underdeveloped embryos at the dispersal time, and thus it is necessary to promote embryo growth to facilitate germination. *Pseudonarcissus* also exhibits physiological dormancy (PD), which must be overcome with several months of warm and/or cold stratification. This dormancy class is known as morphophysiological dormancy (MPD) and it is considered the most difficult to overcome [16]. Thus, although all taxa in the section *Pseudonarcissus* have a high capacity for vegetative propagation from bulbs, germination of their seeds is a complex problem [17]. Indeed, both Vandelook et al. [10] and Newton et al. [11] highlighted the difficulty of germinating *Narcissus pseudonarcissus* seeds. Furthermore, three levels of MPD have been described in section *Pseudonarcissus* [12,13,14], and thus further detailed study is necessary.

In the present study, we investigated seed germination in *N. pseudonarcissus* subsp. *munozii-garmendiae* (Fern. Casas) Fern. Casas (referred to as *N. munozii-garmendiae* in the following) and *N. pseudonarcissus* subsp. *nevadensis* (Pugsley) A. Fern. (referred to as *N. nevadensis* in the following). Both taxa have very limited geographical distributions and small populations that are sensitive to habitat perturbations. Therefore, they are considered threatened and are included in the Red List of Spanish Vascular Flora [18]. Understanding their germination processes is essential for *ex-situ* plant production for medicinal applications of their alkaloid contents, as well as for boosting the wild plant populations and ornamental uses (trumpet daffodils), thereby avoiding the damaging effects on wild populations caused by the large-scale extraction of bulbs. In addition, maximizing their genetic diversity via sexual reproduction will facilitate genetic improvements in the genus to allow increased production of the alkaloids required by the pharmaceutical industry. In addition, the long-term storage of seeds may be possible under low temperature and moisture conditions, thereby providing the advantage of storing large amounts of live genetic plant material in a very small space.

**Figure 1:**
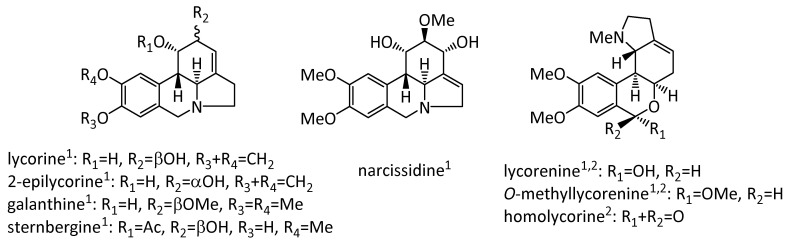
Alkaloids isolated from *N. nevadensis*^1^ [19] and *N. muñozii-garmendiae*^2^ [20].

According to Berkov et al. [7], both *N. munozii-garmendiae* and *N. nevadensis* have high alkaloid contents that typically comprise more than 0.1% of their dry weight in the bulbs, leaves, and flowers. Some of the specific alkaloids isolated from these taxa are shown in Figure 1.

Evidence indicates that lycorine has strong pharmacological effects on many diseases, including anti-leukemia, anti-tumor, anti-angiogenesis, antiviral, antibacterial, anti-inflammatory, and antimalarial effects, as well as exhibiting very low toxicity and mild side effects [21]. In addition, galanthine is active against various tumor cells and as a hypotensive. Galanthine also has a powerful cholinergic activity, and thus it has attracted much interest for use in the treatment of myasthenia gravis, myopathy, and diseases of the central nervous system [22]. Sternbergine possesses antiparasitic and antiplasmodial activities [23], and narcissidine is an effective acetylcholinesterase inhibitor in terms of both its activity and selectivity [24]. Lycorenine, *O*-methyllycorenine, and homolycorine are moderately active in inhibiting the in vivo and in vitro growth of various tumor cells. Furthermore, homolycorine possesses a high antiretroviral activity and it has a hypotensive effect on arterial pressure, while lycorenine has a vasodepressor action and analgesic activity [22].

Therefore, there is much medical interest in both subspecies, so it is necessary to study their propagation techniques using seeds. The main aim of this study was to examine whether the morphological differences between these two phylogenetically closely related taxa in the genus *Narcissus* are associated with differences in seed dormancy and germination characteristics. In particular, we analyzed the following characteristics:

(a) Phenology of embryo growth, dormancy breakage, and radicle emergence
(b) Phenology of seedling emergence
(c) Effects of temperature on embryo growth
(d) Effect of gibberellic acid (GA_3_) on embryo growth
(e) Effects of different stratification and incubation temperatures on germination
(f) Induction of dormancy by extreme temperatures
(g) Temperature requirements for shoot emergence in *N. munozii-garmendiae*

## 2. Results

### 2.1. Outdoor Experiments

#### 2.1.1. Phenology of Embryo Growth, Dormancy Breakage, and Radicle Emergence

- *N. munozii-garmendiae*

**Figure 2:**
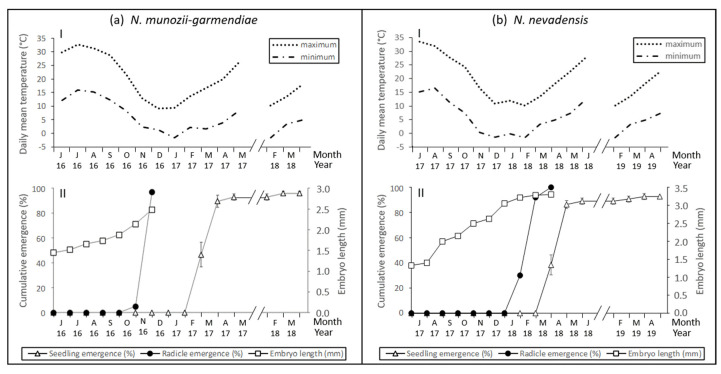
Phenology curves for: (**a**) *N. munozii-garmendiae* seeds sown in soil in an unheated shade house during June 2016; and (**b**) *N. nevadensis* seeds sown during July 2017. The daily mean minimum and maximum air temperatures (I) and phenology of the embryo length, root emergence, and seedling shoot emergence (II) are shown in (**a**,**b**).

At the beginning of the seed burial experiment on June 1, 2016, the mean embryo length was 1.45 ± 0.03 mm. Embryos grew slowly between this date and October 1 when the mean embryo length was 1.88 ± 0.04 mm. During this period, the mean maximum and minimum daily temperatures were 30.7 °C and 13.9 °C, respectively (Figure 2a(I)).

**Figure 3:**
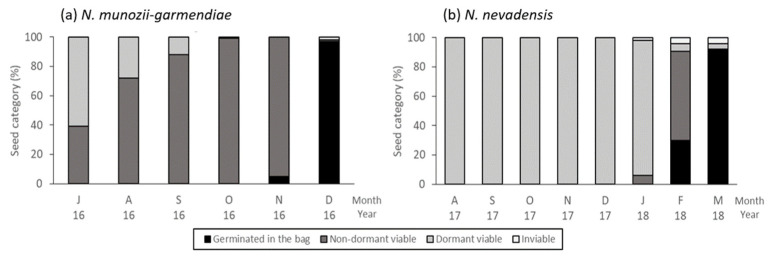
Changes in the percentages of dormant, non-dormant, non-viable, and germinated seeds: (**a**) *N. munozii-garmendiae* seeds buried during June 2016 and exhumed each month for 6 months; and (**b**) *N. nevadensis* seeds buried during July 2017 and exhumed each month for 8 months.

However, the embryos grew more rapidly between October 1 and November 1 when the mean maximum and minimum temperatures were 21.6 °C and 8.4 °C, respectively. Thus, the embryo length was 2.14 ± 0.05 mm on November 1 when 5% of the seeds had an emerged radicle and the remaining 95% had overcome dormancy (Figure 2a and Figure 3a). Between November 1 and December 1, when the maximum and minimum temperatures were 1.3 °C and 2.3 °C, respectively, the embryo length increased from 2.14 mm to 2.49 ± 0.01 mm (close to 2.52 mm, which is the critical length). The seed radicle emergence rate was 97% (Figure 2a(II) and Figure 3a). Embryo growth and the emergence of all radicles occurred the during autumn months of October and November.

- *N. nevadensis*

At the beginning of the seed burial experiment on 1 July 2017, the mean embryo length was 1.33 ± 0.05 mm and the embryos grew little during July. Between August 1 and December 1, embryo growth was continuous from 1.40 ± 0.03 mm to 2.62 ± 0.06 mm. During this period, the mean maximum and minimum daily temperatures were 25.2 °C and 9.0 °C, respectively (Figure 2b(I)). Embryo growth was more rapid between 1 December 2017 and 1 March 2018, when the mean maximum and minimum temperatures were 11.0 °C and –1.0 °C, respectively. On 1 February 2018, the mean embryo length was 3.22 ± 0.06 mm and 30% of the seeds had germinated within the bags, where 61% of the seeds had already overcome dormancy. On 1 March 2018, the embryo length was 3.28 ± 0.01 mm (close to the critical length of 3.32 mm) and the seed radicle emergence rate was 92% (Figure 2b(II) and Figure 3b). Radicle emergence in all seeds occurred during the winter months of January and February.

#### 2.1.2. Phenology of Seedling Emergence

- *N. munozii-garmendiae*

Seedling emergence did not begin until the middle of February 2017 despite the fact that radicle emergence occurred in 97% of the seeds by December 1, 2016. The cumulative seedling emergence rate was 46.67% on 1 March 2017 and 93% on 1 May 2017. No additional seedling emergence occurred until 1 April 2018, when the rate increased to 96%. Subsequently, no further seedlings emerged (Figure 2a(II)).

- *N. nevadensis*

There was almost no delay between radicle emergence and seedling emergence. On 1 March 2018, the radicle had emerged in 92% of the seeds. The cumulative seedling emergence rate was 38% on 1 April 2018, 86% on 1 May 2018, and 89% on 1 June 2018. Subsequently, no seedlings emerged until late winter–early spring during 2019. The final cumulative seedling emergence rate was 92.7 ± 2.4% (Figure 2b(II)).

### 2.2. Laboratory Experiments

#### 2.2.1. Effects of Temperature on Embryo Growth

- *N. munozii-garmendiae*

The mean embryo length in seeds cold stratified (5 °C) in the light for 90 days was 1.58 ± 0.03 mm (Table 1), which was slightly higher than the mean in freshly matured seeds (1.45 ± 0.03 mm), and no embryo achieved the minimum embryo length (2.20 mm) required for germination. The growth of embryos was also very slow and no embryo achieved the minimum length when cold-stratified seeds were transferred to 15/4 °C in the light or darkness for 45 days.

Embryos from seeds that were warm stratified for 90 days reached a mean length of 1.95 ± 0.03 mm at 25/10 °C and 1.86 ± 0.03 mm at 28/14 °C. When these seeds were transferred to 15/4 °C in the darkness for 45 days, the germination percentages were 100% and all embryos reached the critical length.

- *N. nevadensis*

After cold stratification for 120 days in the light at 5 °C and subsequent transfer to 15/4 °C, the embryo length was 2.77 ± 0.13 mm and 52% of the seeds germinated, where 56% of the embryos reached the minimum length (2.8 mm), whereas the results were 3.24 ± 0.03 mm, 64%, and 100%, respectively, when the seeds were incubated at 15/4 °C for 30 days but after treatment A (autumn + winter temperatures). Temperatures of 20/7 °C and 28/14 °C were not effective for promoting embryo growth and germination (Table 2).

The embryo growth and germination percentage results obtained in the dark were similar to those obtained in the light, but the final values were higher for both parameters. After cold stratification (5 °C) for 120 days followed by incubation at 15/4 °C (treatment B), the mean embryo length was 3.08 ± 0.11 mm and 72% of the seeds germinated. For the seeds incubated at 15/4 °C for 30 days after treatment A, the mean embryo length was 3.28 ± 0.02 mm and 96% of the seeds germinated (Table 2).

#### 2.2.2. Effect of GA_3_ on Embryo Growth

- *N. munozii-garmendiae*

GA_3_ did not stimulate embryo growth or germination. After 60 days at 15/4 °C, the embryo length in seeds imbibed in GA_3_ solution was 1.64 ± 0.03 mm in the light and 1.72 ± 0.04 mm in darkness, which were similar to the lengths of seeds imbibed in distilled water, i.e., 1.62 ± 0.03 mm and 1.69 ± 0.03 mm, respectively. No germination occurred in all cases (data not shown).

- *N. nevadensis*

GA_3_ did not stimulate embryo growth or promote germination. After 60 days at 15/4 °C, the embryo length in seeds imbibed in GA_3_ solution was 2.11 ± 0.03 mm in the light and 2.18 ± 0.06 mm in darkness, which were similar to the values obtained for seeds imbibed in distilled water, i.e., 2.03 ± 0.05 mm and 2.14 ± 0.06 mm, respectively. No germination occurred in all cases (data not shown).

#### 2.2.3. Effects of Temperatures of Stratification and Incubation on Germination

- *N. munozii-garmendiae*

**Figure 4:**
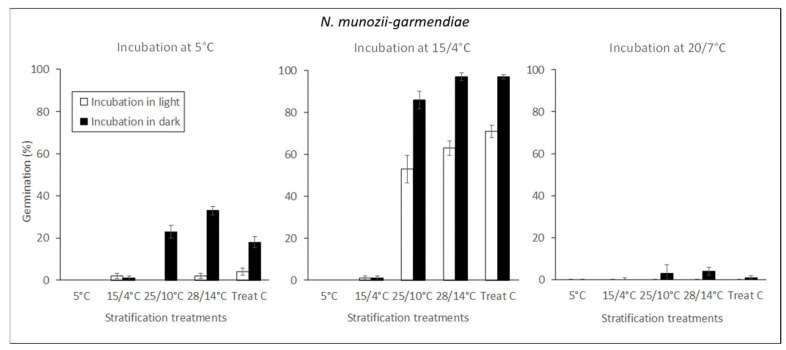
Germination percentages (mean ± standard error, standard error > 2%) for *N. munozii-garmendiae* seeds at different incubation temperatures (5, 15/4, and 20/7 °C) following different 90-day stratifications (5, 15/4, 25/10, 28/14 °C, and treatment C). White and black bars denote the germination percentages after incubation in the light and darkness, respectively.

Seeds that were cold stratified (5 °C) for 90 days did not germinate when they were transferred to different incubation temperatures (Figure 4). By contrast, warm-stratification treatments for 90 days at 25/10 °C, 28/14 °C, or a combination of high temperatures (i.e., treatment C: 28/14 °C (30 days) + 25/10 °C (30 days) + 20/7 °C (30 days)) were highly effective at breaking dormancy. In particular, the percentage of radicle emergence was higher than 86% following incubation at 15/4 °C in darkness.

The optimum incubation temperature was 15/4 °C, followed by 5 °C and 20/7 °C. No seed germination occurred above these temperatures (data not shown). The germination percentages were significantly higher in darkness than the light.

- *N. nevadensis*

**Figure 5:**
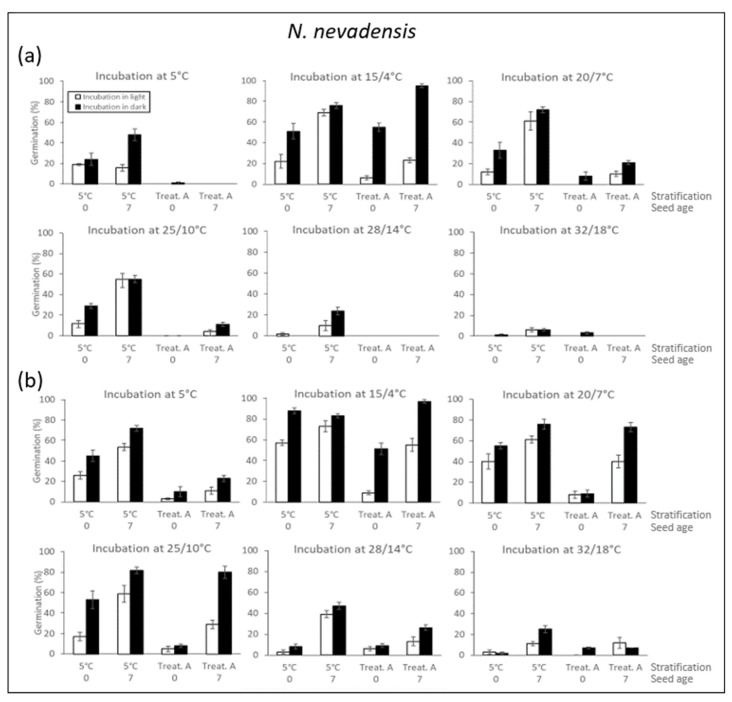
Germination percentages (mean ± standard error, standard error >2%) for *N. nevadensis* seeds at different incubation temperatures (5, 15/4, 20/7, 25/10, 28/14, and 32/18 °C) and ages (0 and 7 months) following different 120-day stratifications (5 °C and treatment A). (**a**) Stratification in light. (**b**) Stratification in darkness. White and black bars represent the germination percentages during incubation in light and darkness, respectively.

In seeds aged 0 months that were cold stratified (5 °C) for 120 days in darkness, 88% of the seeds germinated after incubation at 15/4 °C in darkness. After 5 °C, 20/7 °C, and 25/10 °C in darkness, the germination percentages ranged between 45% and 53%. The germination percentages were lower for the seeds incubated in the light than the darkness and the highest value was 57% at 15/4 °C (Figure 5).

For seeds stratified under treatment A (20/7 °C (30 days) + 15/4 °C (30 days) + 5 °C (60 days)) in both the light and darkness, and then incubated at all of the different temperatures investigated, the germination percentages were lower compared with the cold-stratified seeds. The highest germination percentage was obtained for seeds incubated at 15/4 °C in darkness following treatment A in the light (Figure 5).

The germination percentages increased with the seed age, as observed for the 7-month-old seeds cold stratified for 120 days and then incubated at 5 °C, 20/7 °C, and 25/10 °C in both the light and darkness. This positive effect of seed age on germination was marked after treatment A in darkness, and the differences between germination at 5 °C, 15/4 °C, and 20/7 °C were significant in the light and darkness. The results showed that 97% of the seeds germinated at 15/4 °C in darkness and 80% at 25/10 °C in darkness (Figure 5).

#### 2.2.4. Induction of Dormancy by Extreme Temperatures

- Effect of low temperature (5 °C) on *N. munozii-garmendiae*

For seeds incubated at 28/14 °C for 90 days, subsequently transferred to 25/10 °C or 20/7 °C for 45 days, and then incubated at 15/4 °C for 45 days, the percentage germination was ≥94% and the embryos grew to ≥2.47 mm (Table 3). By contrast, among seeds transferred from 28/14 °C to 5 °C and then incubated at 15/4 °C, only 4% germinated and the embryos grew to 1.95 mm. Thus, exposure to 5 °C induced secondary dormancy in most seeds.

- Effect of summer temperature (32/18 °C) on *N. nevadensis*

In the control seeds (4 months at 5 °C), the embryos grew to 3.10 mm after incubation for 30 days at 15/4 °C in darkness and 82% of the seeds germinated (viability = 100%). However, the embryos only grew to 2.70 mm and only 5% of the radicles emerged (treatment 3, Table 4) when the seeds were exposed to 32/18 °C for 30 days in the middle of the cold stratification period, before then incubating at 15/4 °C in darkness. After exposure to 32/18 °C, a further 90-day or 120-day cold stratification period was required to reach a similar embryo size or germination percentage to those obtained under the control treatment (Table 4). The seed viability (99%) was not affected.

#### 2.2.5. Temperature Requirements for Shoot Emergence in *N. munozii-garmendiae*

**Figure 6:**
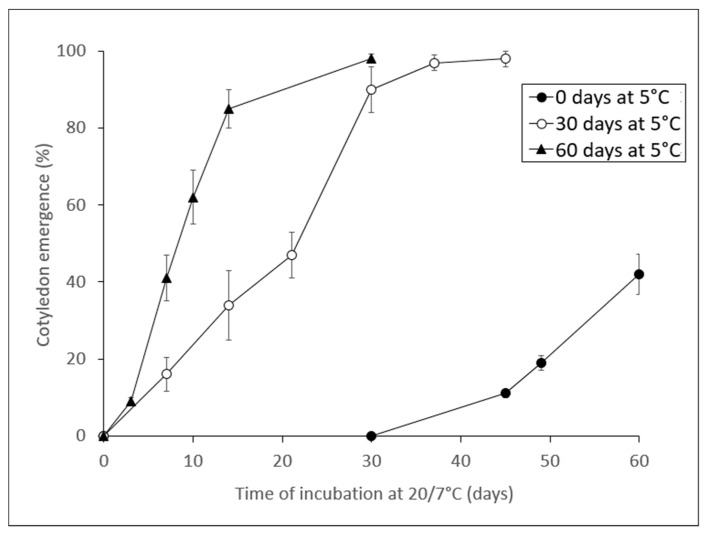
Cotyledon emergence in germinated *N. munozii-garmendiae* seeds at 20/7 °C without or with cold stratification at 5 °C for 30 days or 60 days.

Cold temperature (5 °C) promoted shoot emergence from seeds with emerged radicles. Consequently, prolonging the cold pre-treatment period shortened the incubation time required for development. Thus, shoot emergence occurred in 90% of cold-stratified seeds after only 30 days at 20/7 °C following 30 days at 5 °C (total period = 60 days). Among seeds that were not cold-stratified, shoot emergence only occurred in 42% after 60 days at 20/7 °C (Figure 6). Following stratification for 60 days at 5 °C, shoot emergence occurred in 85% of seeds after incubation for only 14 days at 20/7 °C.

## 3. Discussion

The main aim of the present study was to assess the germination requirements for two subspecies of *N. pseudonarcissus* that produce alkaloids with potential pharmaceutical applications. Determining this information will facilitate the development of detailed protocols for producing plants from seeds and allow the different germination requirements to be analyzed in these close taxa (see Conclusions).

The results obtained in the present investigation and previous studies [10,12,13] clearly demonstrate that a protocol for producing plants from seeds cannot be generalized to the entire *Narcissus* genus because the adaptations of different taxa to their natural habitats determine their germination characteristics. Furthermore, the germination strategy differs among subspecies of *N. pseudonarcissus*, with different levels of MPD according to the results obtained in the present study.

It is essential to specify the level of MPD for the seeds of these species to facilitate their germination by breeders and hybridizers because problems such as unsuccessful cultivation or slow regeneration make it difficult to meet the increasing pharmaceutical demands for these drugs [3]. In addition, the use of seeds allows the production of “virus-free” plants because most *Narcissus* viruses are not seed-borne [9].

After the seeds of *N. munozii-garmendiae* were exposed to warm stratification (25/10 or 28/14 °C) for 90 days, followed by cool temperatures (15/4 °C in darkness) for 45 days, the embryos grew and the radicles then emerged from the seeds. However, the embryos grew little and no seeds germinated when they were first cold stratified at 5 °C for 90 days and then incubated at 15/4 °C in darkness (Table 1). Hence, embryo growth (i.e., loss of morphological dormancy (MD)) occurred during exposure to warm and cool temperatures. This response indicates that the seeds of *N. munozii-garmendiae* exhibit some level of simple MPD [16]. Embryo growth started after the seeds were exposed to favorable temperatures, thereby indicating that MD and PD were overcome simultaneously. These findings indicate that the MPD level was not non-deep simple MPD because PD is broken first and the embryos then grow rapidly (MD) in this MPD level [16]. The fact that GA_3_ did not stimulate embryo growth and germination also suggests that this was not the appropriate MPD level. In addition, intermediate and deep simple MPD could be excluded because after embryo growth, the seeds did not require cold exposure for the radicle to emerge (Figure 2a). Deep simple double MPD was excluded because shoot emergence only occurs after the second winter in this level [25].

The embryo growth patterns observed in laboratory trials were supported by the phenological tests under closed natural conditions in the unheated frame shade house (Figure 2a). Thus, the high summer temperatures provided appropriate conditions for embryo growth and radicle emergence occurred as the temperature declined in the autumn. However, shoot emergence only occurred three months later after the seeds with emerged radicles were subjected to low winter temperatures. The delay between radicle emergence and shoot emergence is known as epicotyl MPD, where the breakage of PD in the root occurs in response to a sequence of warm and cool temperatures, thereby indicating that this was nondeep MPD. The difference between these MPD levels is that non-deep simple epicotyl MPD does not require cold stratification to break PD for the shoots, as found in *Viburnum odoratissimum* [26], whereas the opposite occurs in seeds with deep simple epicotyl MPD. In *N. munozii-garmendiae*, cold stratification of seeds with emerged radicles increased the rate of shoot emergence when these seeds were exposed to spring (20/7 °C) temperatures (Figure 6). Thus, in non-cold stratified seeds, the shoot emergence rate only reached 42% after 60 days at 20/7 °C, whereas the rate was up to 90% when seeds with emerged radicles were cold stratified for 30 days and then incubated at 20/7 °C during 30 days (total time 30 + 30 = 60 days). Therefore, we conclude that *N. munozii-garmendiae* seeds are characterized by deep simple epicotyl MPD.

In *N. nevadensis*, the highest embryo length and radicle emergence percentage occurred in seeds that were cold stratified (5 °C) for 4 months, or in seeds stratified at autumn + winter temperatures (20/7 °C (30 days) + 15/4 °C (30 days) + 5 °C (60 days)) and then incubated at 15/4 °C for 30 days. However, stratification at 20/7 °C or 28/14 °C was not effective for overcoming dormancy (Table 2). In tests where seeds were stratified in cold conditions (5 °C) for 120 days in darkness (Figure 5b) and then incubated at 5 °C for 30 days, the germination rates were 45% and 72% with seed ages of 0 and 7 months, respectively. Therefore, cold stratification was the only requirement for embryo growth and radicle emergence, and thus these seeds must be characterized by one of three levels of complex MPD. The fact that cold stratification was the only requirement for dormancy breakage, embryo growth, and germination, and that GA_3_ could not substitute for cold stratification indicates that the seeds of *N. nevadensis* were characterized by deep complex MPD [27,28]. However, some of the results obtained in the present study did not agree well with this MPD level, i.e., the positive effects of dry storage under laboratory conditions (i.e., seed age) (Figure 5b) and exposure to slightly warm stratification (20/7 °C (30 days) + 15/4 °C (30 days)) on the germination percentage (Table 2). These characteristics are typical of intermediate PD in seeds with fully-developed embryos, which are often (but not always) sensitive to GA_3_ [29]. These results suggest that the PD is at an intermediate level in *N. nevadensis* and that the seeds exhibit intermediate complex MPD, as described in the close taxa *N. alcaracensis* [13].

The presence of two different germination modes comprising deep simple epicotyl MPD in *N. munozii-garmendiae* and intermediate complex MPD in *N. nevadensis* confirms that the germination responses to environmental factors can be precise mechanisms related to habitat choice in plants because the environmental conditions in a specific habitat only allow dormancy breakage and germination in particular species. Thus, very closely related species in the genus *Narcissus* that occupy different habitats may have different germination strategies, but also different alkaloid contents [6,7] because they comprise chemical defenses developed as an evolutionary response to the existing pressures in a habitat, such as predators or pests [30].

An important point to consider when producing these subspecies is that the seeds of *N. munozii-garmendiae* and *N. nevadensis* had higher percentage germination rates in darkness compared with light conditions, as also found in other species that belong to the *Pseudonarcissus* section of *Narcissus* [12,13,14,31,32]. Photoinhibition is expected to occur mainly in seeds with intermediate seed sizes of 1–27 mg [33], as found in both *N. munozii-garmendiae* and *N. nevadensis*. Another trait that may affect the response of seeds to light is the pigmentation of the seed coat, which influences both the spectral composition and quantity of light transmitted [34]. Photoinhibition is related to black or dark colored seed [33,35] and it is more frequent in certain lineages, such as in monocot taxa in the order Asparagales, which includes the family Amaryllidaceae [33].

The results obtained in the dormancy induction tests are also interesting because the two taxa exhibited opposite responses. Thus, low temperatures made the *N. munozii-garmendiae* seeds re-enter secondary dormancy, whereas high temperatures had this effect on *N. nevadensis*. This information is very important for the plant production process from seeds because inadequate storage at extreme temperatures can lead to re-entry into dormancy and delay production.

In a recent review of the genus *Narcissus* in the Iberian Peninsula, Aedo [15] considered both of the taxa analyzed in the present study as subspecies of *Narcissus pseudonarcissus*. However, our results support the existence of physiological differences between these two subspecies according to their different requirements for breaking dormancy and germination. These differences may be associated with their morphological variations and different alkaloid composition [7,19,20], and it is possible that *N. munozii-garmendiae* and *N. nevadensis* may be treated as two closely related species.

## 4. Materials and Methods

### 4.1. Plant Material and Seed Sources

#### 4.1.1. Narcissus Munozii-Garmendiae

*N. munozii-garmendiae* flowers early in February and each floral scape produces a single capsule containing 30–40 seeds. The seeds mature in late April–early May. The capsules become dark yellow when ripe and they open rapidly to release seeds. The seeds were collected in Sierra Madrona (central–south Spain, 30SUH8356, at 830 m a.s.l.).

Capsules were collected in four consecutive years on 4 May 2015, 2 May 2016, 28 April 2017, and 3 May 2018, when 100, 300, 150, and 100 capsules were acquired, respectively. The capsules were kept in laboratory trays to allow seed release in a natural manner. The released seeds were desiccated in the laboratory at room temperature (22 °C, 40–50% RH) from the collection date until June 1 (seed age = 0 months). The desiccated seeds were then kept in paper envelopes until the experiments commenced.

In the freshly matured seeds, the embryo length (E) was 1.45 ± 0.03 mm and the seed size (S) was 3.24 ± 0.06 mm (mean ± standard error, n = 25, E:S = 0.45), thereby suggesting that the embryo was underdeveloped. The small embryo and lack of germination after 30 days of incubation at temperatures found in the natural habitat during the course of the year (5 °C, 15/4 °C, 20/7 °C, 25/10 °C, 28/14 °C, and 32/18 °C) indicated that the *N. munozii-garmendiae* seeds exhibited some level of MPD. Preliminary experiments with seeds collected in 2015 showed that seeds germinated after 3 months of warm stratification (28/14 °C) followed by incubation at cool temperatures (15/4 °C).

#### 4.1.2. Narcissus Nevadensis

*N. nevadensis* flowers from early April to May and each floral scape produces 1–3 capsules containing 35–45 seeds/capsule. The seeds mature from early to mid-June. The mature capsules become light brown and a quarter of their length opens at the apical end, and thus most of the seeds are retained inside for 3–4 weeks. The seeds were collected in the Meridional Iberian System (central–east Spain, 30TWK7227, at 930 m a.s.l.).

Capsules were collected on 6 June 2015, 4 June 2016, 5 June 2017, and 18 June 2018, when 200, 100, 300, and 250 capsules were acquired, respectively. The released seeds were kept in laboratory trays until July 1 to complete their desiccation and the seed age was considered to be 0 months on this date. The seeds were then stored in paper envelopes at room temperature until the beginning of the experiments.

In the freshly matured seeds, the embryo length was 1.33 ± 0.05 mm and the seed size was 3.93 ± 0.06 mm (mean ± standard error, n = 25, E:S = 0.34), thereby indicating that the embryo was underdeveloped. The small embryo and lack of germination during 30 days of incubation at a wide range of temperatures strongly suggested that the *N. nevadensis* seeds exhibited some level of MPD. Preliminary studies with seeds collected in 2015 showed that the seeds germinated after incubation at cool temperatures (15/4 °C) following 4 months of cold stratification (5 °C) or 4 months of low heat + cold stratification.

### 4.2. Outdoor Experiments

Outdoor experiments were conducted to determine the timings of the main events in the seed/seedling stage during the life cycles of *N. munozii-garmendiae* and *N. nevadensis* relative to the seasonal temperature cycle. The seeds were kept under near natural temperature conditions in a non-heated metal frame shade house located in an experimental field (at 690 m a.s.l.) at the Technological School of Agronomy and Forestry in Albacete (southeast Spain) at 135–260 km from the collection sites. The air temperature in the shade house was recorded continuously with a data logger to obtain the monthly average maximum and minimum temperatures.

The growing medium used in the pots and trays containing seeds comprised a mixture of sterilized peat and sand (2:1 v/v). To simulate the humidity conditions in the soil in the natural habitat, a water control system was programmed to water to the field capacity once a week, but it was reduced to twice a month in July and August in order to simulate the summer drought that commonly occurs in the Mediterranean area. In addition, water was withheld when the substratum was frozen in winter.

#### 4.2.1. Phenology of Embryo Growth, Dormancy Breakage, and Radicle Emergence

The experiment commenced when the seed age was 0 months in both taxa, i.e., 1 June 2016 for *N. munozii-garmendiae* and 1 July 2017 for *N. nevadensis*. The difference was due to the low amount of *N. nevadensis* seeds produced in 2016. For each species, 10 groups each comprising 100 seeds were mixed with fine-grain sterilized sand. Each group of seeds was placed in a fine-mesh polyester cloth bag and buried at a depth of 5 cm in a pot. The pots were placed in the shade house. The bags were exhumed each month starting from 1 month after the experiment commenced, and the contents were sieved (1-mm mesh) to separate the seeds from the sand. We recorded the percentages of seeds with emerged radicles, and the mean embryo length was determined by excising the embryos from 25 healthy-looking seeds and measuring their lengths using an ocular micrometer. The embryo length was recorded as the critical embryo length in seeds with emerged radicles.

The critical embryo length for radicle emergence is the length of the embryo at the time the seed coat splits but before the radicle emerges [10]. In *N. munozii-garmendiae*, the critical embryo length was 2.52 ± 0.05 mm (mean ± standard error, n = 25, range = 2.2–3.1 mm). The 25 seeds tested to calculate the critical embryo length received warm (28/14 °C) plus cool (15/4 °C) stratification treatments. The minimum value for this range (2.2 mm) corresponds to the minimal value required for radicle emergence and reaching this length is a good indicator that dormancy has been overcome [12]. In *N. nevadensis*, the critical embryo length was 3.32 ± 0.05 mm (range 2.8–4 mm). The 25 seeds tested to calculate the critical embryo length received cold (5 °C) plus cool (15/4 °C) stratification treatments.

Non-germinated exhumed seeds and those not used for embryo length measurements were incubated for 30 days at 15/4 °C in darkness. After incubation, the following seed status percentages were calculated: (1) seeds with emerged radicles within the bag; (2) viable non-dormant seeds (i.e., those that germinated in the incubation phase at 15/4 °C); (3) viable dormant seeds (i.e., those that did not germinate at 15/4 °C, but with healthy embryos); and (4) non-viable seeds (i.e., those with a rotten appearance or containing a dead embryo after excision).

#### 4.2.2. Phenology of Seedling Emergence

On 1 June 2016 for *N. munozii-garmendiae* and 1 July 2017 for *N. nevadensis*, three trays (20 cm × 30 cm × 8 cm) with drainage holes were filled with the growing medium. One-hundred seeds were sown in each tray at a depth of 5 mm and equidistant from each other to avoid contact. The trays were placed in the shade house. During the 2 years after sowing, the seed trays were examined once each week and seedlings were counted and removed.

### 4.3. Laboratory Experiments

#### 4.3.1. General Conditions

Experiments were conducted in chambers with controlled temperature and light regimes (Ibercex model F-4, Madrid, Spain), which were equipped with a digital temperature and light control system (0.1 °C, cool white fluorescent light, 25 mol m^−2^ s^−1^ (1350 lux)). Seeds were tested for radicle emergence in a 12 h daily photoperiod (light) and in continuous darkness (darkness), which was achieved by wrapping Petri dishes in a double layer of aluminum foil, and incubating at a constant temperature of 5 °C, and 12/12 h alternating temperature treatments, where the higher temperature coincided with the light phase and the lower temperature with darkness. Seeds were incubated on two layers of filter paper moistened with distilled water in Petri dishes with a diameter of 9 cm. The dishes were sealed with Parafilm to minimize the loss of water. The alternating temperature regimes simulated the mean maximum and mean minimum monthly temperatures during the annual climate cycle in continental inland areas of Iberian Peninsula, where 15/4 °C corresponded to November and March, 20/7 °C to October and April, 25/10 °C to September and May, 28/14 °C to June and August, and 32/18 °C to July. The 5 °C treatment simulated the mean temperature recorded during the winter months of December, January, and February [36].

The percentage germination (radicles emerged ≥ 1 mm and clearly visible) was calculated based on the number of apparently viable seeds. Non-germinated seeds were checked for viability based on the appearance of the embryo, particularly the color and turgidity. Seeds were considered viable if the embryo was white and resistant to a slight pressure applied with tweezers. These indicators of seed viability agreed closely with the results obtained using the tetrazolium test [37].

#### 4.3.2. Effects of Temperature on Embryo Growth

Experiments were conducted to determine the optimal temperature for embryo growth.

- *N. munozii-garmendiae*

On 1 June 2016, three groups each comprising 200 seeds were placed on two moistened layers of filter paper in three Petri dishes measuring 9 cm. Each Petri dish was stratified in light for 90 days under one of the following temperature conditions: 5 °C, 25/10 °C, and 28/14 °C. Each month, 25 seeds were sampled from each temperature treatment to measure the embryo lengths, where the mean and standard error were calculated for each sample. In addition, after stratification for 90 days at the specified temperatures, two samples of 25 seeds were transferred from each Petri dish to 15/4 °C for 45 days, where one sample was kept in light and the other in darkness. At the end of this period, the mean embryo length and final germination percentage were calculated for each sample.

- *N. nevadensis*

On 1 September 2016, eight Petri dishes each containing 200 seeds on two moistened layers of filter paper were stratified for 120 days: four in light and four in darkness. For each illumination condition, four Petri dishes were tested under each of the following four temperature treatments: 5 °C, 20/7 °C, 28/14 °C, and treatment A (20/7 °C (30 days) + 15/4 °C (30 days) + 5 °C (60 days)). Each month during the stratification period, 25 seeds were sampled from each Petri dish and the embryos were measured after excision. In addition, at the end of the 120-day period, 25 seeds were transferred from each of the eight stratification conditions and incubated at 15/4 °C for 30 days (treatment B). The light conditions were the same during stratification and incubation. The embryo length and germination percentage were measured.

#### 4.3.3. Effect of GA_3_ on Embryo Growth

On 1 September 2016, for each subspecies, four groups each comprising 50 seeds were placed in Petri dishes with a diameter of 9 cm on two sheets of filter paper, which was moistened with a solution of 1000 mg L^−1^ of GA_3_ in distilled water, and incubated at 15/4 °C, with two dishes in light and two in darkness. After 30 and 60 days, the germination percentage was calculated and 25 embryos were measured in both light and dark conditions. The results were compared with those obtained using seeds incubated under the same conditions but moistened with distilled water alone.

#### 4.3.4. Effects of Stratification and Incubation Temperatures on Germination

- *N. munozii-garmendiae*

On 1 June 2016 (seed age = 0 months), five groups each comprising 800 seeds were placed on sheets of wet filter paper in five Petri dishes with a diameter of 15 cm. The Petri dishes were stratified in light for 3 months at: 5 °C, 15/4 °C, 25/10 °C, 28/14 °C, and treatment C (28/14 °C (30 days) + 25/10 °C (30 days) + 20/7 °C (30 days)), which simulated the conditions during late summer and early autumn in the natural habitat. The stratification treatments were only conducted in light because fungal infections were promoted by prolonged exposure to high temperatures. Hence, it was necessary to clean the seeds with distilled water every 1 or 2 weeks and this repetitive manipulation could have affected the darkness treatment. Following the stratification periods, the seeds were transferred and incubated for 45 days at 5 °C, 15/4 °C, 20/7 °C, and 25/10 °C in light and darkness.

- *N. nevadensis*

On 1 July 2017 (seed age = 0 months), four groups each comprising 1300 seeds were placed on two moistened layers of filter paper in four Petri dishes measuring 15 cm. Two Petri dishes were stratified for 120 days in light and two in darkness because two temperature stratification treatments were tested for each illumination condition: 5 °C (120 days) and treatment A (20/7 °C (30 days) + 15/4 °C (30 days) + 5 °C (60 days)), where treatment A simulated the autumn–winter conditions. Following the stratification treatments, the seeds were incubated for 30 days under 12 different temperature–light conditions (six temperature regimes × two light conditions). In order to determine the influence of seed age on the germination capacity, this experiment was repeated on 1 June 2017 (seed age = 7 months).

#### 4.3.5. Induction of Dormancy by Extreme Temperatures

- Effect of low temperature (5 °C) on *N. munozii-garmendiae*

This experiment was conducted to determine whether the low temperatures that occur naturally during the winter could induce secondary PD in seeds where primary PD was overcome and containing embryos that had begun to grow but not to the fullest extent.

On 1 June 2017 (seed age = 0 months), three groups each comprising 200 seeds were placed in Petri dishes measuring 9 cm to test the effects of two different stratification sequences (warm plus cold, and warm plus warm) in light on germination. First, the three groups were incubated at 28/14 °C for 90 days. Second, one of the groups was transferred to incubate at 5 °C for 45 days, another group at 20/7 °C for 45 days, and the third at 25/10 °C for 45 days. Finally, four 25 seed sample replicates from each group were used to determine the percentage germination and embryo growth at 15/4 °C in light for 45 days.

- Effect of summer temperatures (32/18 °C) on *N. nevadensis*

This experiment aimed (1) to verify whether the interruption of the cold stratification period with 1 month of warm stratification (32/18 °C) prevented dormancy breakage, (2) to test whether dormancy was induced by high temperatures (32/18 °C), and (3) to determine whether seeds with secondary dormancy required a period of cold stratification to break dormancy.

On 1 October 2018 (seed age = 3 months), six Petri dishes each containing 150 seeds were exposed to the six light–stratification treatments described in Table 4. After each treatment, four 25 seed sample replicates were used to determine the percentage germination and embryo growth after 30 days at 15/4 °C in darkness.

#### 4.3.6. Temperature Requirements for Shoot Emergence in *N. munozii*-*garmendiae*

This experiment determined whether cold stratification is required for shoot emergence in radicle-emerged seeds and the necessary period of stratification.

On 1 October 2017, seeds with emerged radicles were placed on two sheets of filter paper moistened with distilled water in Petri dishes measuring 9 cm and incubated in light. Three groups each comprising 100 seeds (four replicates of 25 seeds) with roots measuring 2–3 mm in length were stratified in moist cold conditions (5 °C) for 0, 30, or 60 days. After each cold stratification treatment, the seeds were transferred and incubated at 20/7 °C for 60 days, before calculating the shoot emergence percentage (shoot length > 2–3 mm).

### 4.4. Statistical Analysis

In each subspecies, means and standard errors were calculated for the radicle and shoot emergence percentages, and embryo lengths. The factors analyzed comprised the stratification temperature, incubation temperature, light condition during stratification–incubation, time of stratification, concentration of GA_3_ (0 and 1000 ppm), and seed age. In each experiment, the effects of several factors on both embryo length and germination were analyzed by multifactor analysis of variance using SPSS Statistics v24. Seed germinability was evaluated based on the final cumulative germination percentage among the number of viable seeds. If the effect of a factor was significant, differences were compared with Tukey’s multiple comparison test. The normality (Cochran test) and homoscedasticity (David test) of the data were checked before analyses were conducted. The final cumulative germination percentages were square-root arcsine transformed.

## 5. Conclusions

**Figure 7:**
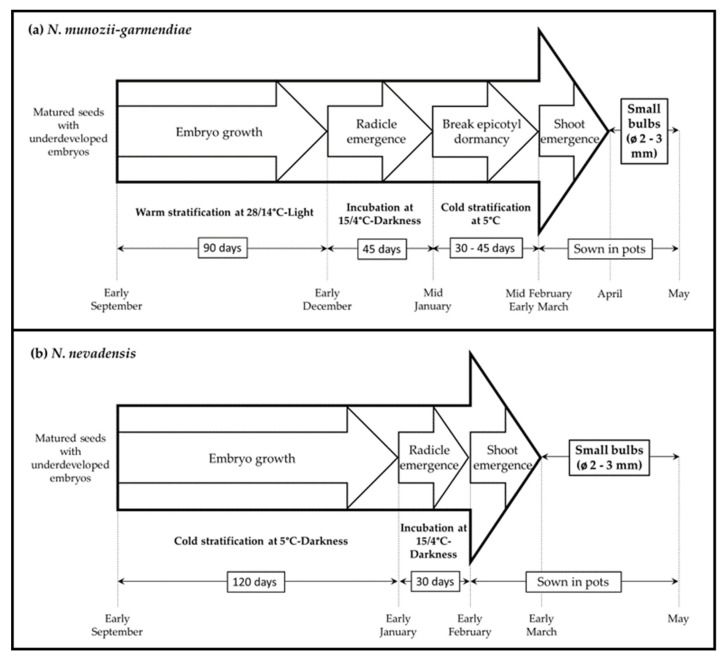
Protocols for plant production from the seeds of *N. munozii-garmendiae* (**a**) and *N. nevadensis* (**b**).

Figure 7 shows the specific protocols developed for producing plants from seeds for each of *N. munozii-garmendiae* and *N. nevadensis*. In both cases, the pots must not be watered from June to October because wet conditions combined with high summer temperatures greatly increase the possibility of bulb decay. In the third plant cycle around February–March, the bulbs will have reached 1.5–2 cm in diameter and they are ready to produce many leaves and flower stems (personal observation). The present exhaustive study elucidated the complexity of seed germination in *Narcissus*, as well as facilitating the use of these clonal lines in gardening and the pharmaceutical industry. These wild taxa can enrich the available genetic variability of *Narcissus* and provide very useful raw materials for breeders and hybridizers.

## Figures and Tables

**Table 1:** Effects of temperature and duration of stratification in the light on the embryo length (mm, mean ± standard error, n = 25) in *N. munozii-garmendiae* seeds. After 3 months of stratification at 5 °C, 25/10 °C, or 28/14 °C, seeds were incubated at 15/4 °C in the light or darkness for 45 days. The seed age at the beginning of the experiment was 0 months. Values followed by different uppercase letters within a column or different lowercase letters within a row are significantly different (*p* < 0.05, Tukey’s multiple comparison test). The first number in parentheses is the percentage of radicle emergence, and the second is the percentage of seeds with an embryo longer than the threshold embryo length required to germinate (2.2 mm).

Previous 90-Day Stratification				Subsequent 45-Day Incubation	
Temperature	30 Days	60 Days	90 Days	15/4 °C Light	15/4 °C Darkness
5 °C Light	1.50 ± 0.03 ^Aa^	1.56 ± 0.03 ^Aab^	1.58 ± 0.03 ^Aab^	1.64 ± 0.03 ^Ab^	1.65 ± 0.03 ^Ab^
	(0, 0)	(0, 0)	(0, 0)	(0, 0)	(0, 0)
25/10 °C Light	1.72 ± 0.03 ^Ba^	1.77 ± 0.03 ^Ba^	1.95 ± 0.03 ^Bb^	2.40 ± 0.03 ^Bc^	2.50 ± 0.00 ^Bc^
	(0, 0)	(0, 0)	(0, 4)	(64, 88)	(100, 100)
28/14 °C Light	1.68 ± 0.03 ^Ba^	1.80 ± 0.02 ^Bb^	1.86 ± 0.03 ^Bb^	2.45 ± 0.03 ^Bc^	2.50 ± 0.00 ^Bc^
	(0, 0)	(0, 0)	(0, 0)	(88, 92)	(100, 100)

**Table 2:** Embryo length (mm, mean ± standard error) in *N. nevadensis* seeds after different periods of stratification and different temperatures in the light and darkness. Treatment A: 20/7 °C (30 days) + 15/4 °C (30 days) + 5 °C (60 days). Treatment B: Incubation at 15/4 °C for 30 days following stratification for 120 days at 5 °C, 20/7 °C, 28/14 °C, and treatment A. Values followed by different uppercase letters within a column or different lowercase letters within a row are significantly different (*p* < 0.05, Tukey’s multiple comparison test). The first number in parentheses is the percentage of radicle emergence and the second is the percentage of seeds with an embryo longer than the threshold embryo length required to germinate (2.8 mm).

Previous 120-Day Stratification								
Duration (days)	Temperatures in Light				Temperatures in Darkness			
	5 °C	20/7 °C	28/14 °C	Treatment A	5 °C	20/7 °C	28/14 °C	Treatment A
30	1.37(0.00 ^Aa^	1.68 ± 0.06 ^Abc^	1.76 ± 0.05 ^Abc^	1.68 ± 0.06 ^Abc^	1.38 ± 0.05 ^Aa^	1.63 ± 0.06 ^Ab^	1.91 ± 0.04 ^Ac^	1.63 ± 0.06 ^Ab^
	(0, 0)	(0, 0)	(0, 0)	(0, 0)	(0, 0)	(0, 0)	(0, 0)	(0, 0)
60	1.50 ± 0.06 ^ABa^	1.77 ± 0.05 ^ABbc^	1.98 ± 0.06 ^ABcd^	1.77 ± 0.07 ^ABbcd^	1.61 ± 0.06 ^ABab^	1.98 ± 0.06 ^Bcd^	2.04 ± 0.04 ^ABd^	1.92 ± 0.09 ^Bcd^
	(0, 0)	(0, 0)	(0, 0)	(0, 0)	(0, 0)	(0, 0)	(0, 0)	(0, 8)
90	1.72 ± 0.05 ^BCa^	1.89 ± 0.05 ^ABab^	2.06 ± 0.0 ^Bbc^	1.92 ± 0.06 ^Bab^	1.88 ± 0.07 ^Bab^	2.20 ± 0.06 ^BCc^	2.10 ± 0.04 ^Bbc^	2.26 ± 0.07 ^Cc^
	(0, 0)	(0, 0)	(0, 4)	(0, 0)	(0, 4)	(0, 4)	(0, 0)	(0, 12)
120	2.02 ± 0.07 ^Ca^	1.96 ± 0.06 ^Ba^	2.14 ± 0.0 ^BCab^	2.45 ± 0.07 ^Cbc^	2.28 ± 0.11 ^Cab^	2.26 ± 0.06 ^Cab^	2.17 ± 0.05 ^BCab^	2.62 ± 0.10 ^Dc^
	(0, 8)	(0, 4)	(0, 4)	(0, 12)	(8, 24)	(0, 12)	(0, 0)	(4, 44)
SUBSEQUENT INCUBATION **(Treatment B)**	2.77 ± 0.13 ^Dbc^	2.26 ± 0.07 ^Ca^	2.32 ± 0.08 ^Ca^	3.24 ± 0.03 ^Dd^	3.08 ± 0.11 ^Dcd^	2.51 ± 0.05 ^Dab^	2.30 ± 0.05 ^Ca^	3.28 ± 0.02 ^Ed^
	(52, 56)	(0, 12)	(0, 8)	(64, 100)	(72, 80)	(0, 16)	(0, 4)	(96, 100)

**Table 3:** Induction of dormancy by cold temperature (5 °C) in *N. munozii-garmendiae* seeds under light conditions. The seed age at the beginning of the experiment was 0 months. Seeds were collected in 2017. Values followed by different uppercase letters within a column are significantly different (*p* < 0.05, Tukey’s multiple comparison test).

Previous Stratification Treatment	Subsequent Incubation	Embryo Length (mm)	Germination (%)
Control: 28/14 °C (90 d)	15/4 °C (45 d)	2.46 ± 0.02 ^C^	88 ± 2.83 ^B^
28/14 °C (45 d) + 5 °C (45 d) + 28/14 °C (45 d)	15/4 °C (45 d)	1.69 ± 0.03 ^A^	0 ± 0.00 ^A^
28/14 °C (90 d) + 5 °C (45 d)	15/4 °C (45 d)	1.95 ± 0.03 ^B^	4 ± 1.63 ^A^

**Table 4:** Induction of dormancy by summer warm temperatures (32/18 °C) and secondary dormancy breakage by cold stratification (5 °C) in *N. nevadensis*. Stratification treatments were conducted in the light. Incubation was applied at 15/4 °C in darkness for 30 days. The table shows the embryo length (mm, mean ± standard error) and germination percentage in each treatment. Values followed by different uppercase letters within a column or different lowercase letters within a row are significantly different (*p* < 0.05, Tukey’s multiple comparison test).

Previous Stratification Treatments	After Stratification	After Incubation Following Stratification	
	Embryo Length (mm)	Embryo Length (mm)	Germination (%)
Treatment 1: Control 5 °C (120 days)	2.64 ± 0.08 ^BCa^	3.10 ± 0.09 ^BCb^	80 ± 1.6 ^D^
Treatment 2: 5 °C (60 days) + 32/18 °C (30 days) + 5 °C (60d)	2.26 ± 0.05 ^Aa^	2.70 ± 0.06 ^Ab^	5 ± 1.0 ^AB^
Treatment 3: Control + 32/18 °C (30 days)	2.61 ± 0.08 ^Ba^	2.82 ± 0.07 ^ABb^	4 ± 1.6 ^A^
Treatment 4: Treatment 3 + 5 °C (60 days)	2.77 ± 0.09 ^BCa^	2.92 ± 0.07 ^ABCa^	11 ± 1.9 ^B^
Treatment 5: Treatment 3 + 5 °C (90 days)	2.80 ± 0.07 ^BCa^	3.01 ± 0.07 ^BCb^	36 ± 3.7 ^C^
Treatment 6: Treatment 3 + 5 °C (120 days)	2.92 ± 0.07 ^Ca^	3.15 ± 0.06 ^Cb^	76 ± 2.3 ^D^
